# Database of Romanian cave invertebrates with a Red List of cave species and a list of hotspot/coldspot caves

**DOI:** 10.3897/BDJ.8.e53571

**Published:** 2020-06-11

**Authors:** Oana Teodora Moldovan, Sanda Iepure, Traian Brad, Marius Kenesz, Ionuț Cornel Mirea, Ruxandra Năstase-Bucur

**Affiliations:** 1 Emil Racovitza Institute of Speleology, Cluj-Napoca, Romania Emil Racovitza Institute of Speleology Cluj-Napoca Romania; 2 Romanian Institute of Science and Technology, Cluj-Napoca, Romania Romanian Institute of Science and Technology Cluj-Napoca Romania; 3 Cavanilles Institute of Biodiversity and Evolutionary Biology, University of Valencia, Spain, Valencia, Spain Cavanilles Institute of Biodiversity and Evolutionary Biology, University of Valencia, Spain Valencia Spain; 4 Emil Racovitza Institute of Speleology, București, Romania Emil Racovitza Institute of Speleology București Romania

**Keywords:** troglobionts, stygobionts, Romania, cave, protection status, vegetation, climate, threats, Red List

## Abstract

**Background:**

The increasing human impact in Romanian caves raises the urgency of publishing a correct database of the strictly-adapted cave fauna. Previous attempts at indexing cave fauna and classifying caves by using their fauna opened many questions regarding the use of an incomplete list of cave species and mixed lists of troglobionts/stygobionts with troglophiles/stygophiles for ranking caves with priority for protection. It has also become obvious that there is a need to publish a list of Romanian cave species that are under threat. Cave species in Romania (and elsewhere) are endemic on small ranges, are unique and must be considered as important units for conservation. A cave must be equally protected if it has one or more rare and strictly endemic cave species. Although not exhaustive, we here provide the first checklist of Romanian troglobionts/stygobionts developed in the framework of the DARKFOOD and GROUNDWATERISK projects, coordinated by the “Emil Racovita” Institute of Speleology, Cluj-Napoca, Romania. The GIS application was used to complement the checklist of cave species with data on caves and surface environments above the caves. Until complete data on species diversity and population sizes are made available for each cave, measures of conservation can be implemented, based on the presence/absence of cave species, while classifications of caves for protection, based on the number of species, must be avoided. We also propose a list of Romanian caves with fauna that are under threat and a tentative Red List of Romanian troglobiont/stygobionts.

**New information:**

This is the first database with identified troglobiont and stygobiont species of Romania, with a critical analysis of their distribution inside the country. A list of caves that need protection for their rare and unique species and a tentative Red List of Romanian cave fauna are also added. A total of 173 species were identified, of which 77 troglobionts and 96 stygobionts are currently registered in 366 caves. The database is divided into two parts, one part with a list of troglobionts, their revised systematic position, cave name, cave code and geographic region; and the second part with the same information on stygobionts. The database represents the contribution of many active researchers, who are the authors of this paper and of review publications of many other authors of the "Emil Racoviță" Institute of Speleology.

## Introduction

The first database containing Romanian cave fauna was the initiative of Emil Racovitza together with René Jeannel, Pierre-Alfred Chappuis and their collaborators as part of the scientific enterprise called *Biospeologica*. In the 7th and 8th series of the *Énumération des grottes visitées*, there are 178 Romanian caves with their cave faunas (Chappuis and Jeannel 1951, Jeannel and Racovitza 1929). Later, Decu and Negrea (1969), Decu and Iliffe (1983), Decu and Racoviță (1994) published reviews on Romanian cave fauna by also including remarks on geographical distribution, origin and evolution. Lists of different cave groups were also published by Decu (1964), Dumitrescu (1979), Dumitrescu (1980), Negrea (1994), Gruia (2003), Tabacaru et al. (2003), Moldovan et al. (2007), Tabacaru and Giurgincă (2013), Giurgincă et al. (2014) etc. The most recent list of invertebrates of Romania including cave species can be found in Moldovan et al. (2007) compiled for the Fauna Europea project. To these contributions are added other papers, not cited here, with published lists on smaller regions of Romania, taxonomic investigations with descriptions of new species and ecological investigations of distinct caves. A recently-published list of cave fauna in Romania, by Nitzu et al. (2016) is a compilation of endemic cave species, both troglo/stygophiles and troglo/stygobionts. The list was used to classify the caves for protection purposes (Nitzu et al. 2018), a classification that introduced serious errors with possible consequences for the integrity of caves and their fauna. The errors were discussed by Moldovan and Brad (2019) and consisted of: quantitative ranking of caves, based on an incomplete and incorrect inventory of cave species, on assumed regional endemic species, stating that troglobionts and troglophiles are equal in setting protection priorities for caves, assuming that known frequency is a true estimator of endemicity, ignoring species that should be on a Red List of Romanian cave species and ignoring the legislation in force that protects cave habitats and not cave species.

Here, we present an updated checklist of Romanian troglobionts and stygobionts, identified to species level. To the bibliographic data, we added our own database and collections inventory. We are aware that the species list will change over time because of advances in taxonomic works: new species are constantly being described and added to the list and taxonomic revisions of certain groups are constantly changing. However, there is an urgent need to provide the first list of troglobionts/stygobionts from Romanian caves for the increased use of caves and above-surface habitats, such as caving for wild tourism, deforestation, surface pollution, all with significant consequences on the subterranean habitats. The database is dynamic and will be continuously updated and corrected.

## General description

### Purpose

The increased human pressure on karst areas and caves in Romania called for a complete list of troglobionts and stygobionts, all endemic for small regions within the country. None of the strictly endemic cave species is protected by the national or European legislation. The only legislative measure that can be referred to, but is almost always ignored, is the Habitats Directive of the European Commission where cave habitats not open to the public are protected (European Habitats Directive 43/92, H 8310). It is impossible to implement feasible protection measures for the 12,000 caves in Romania (Bleahu 2019). However, the identification of caves with particular troglobionts/stygobionts will ease decisions on vulnerable caves and the adoption of appropriate measures for their protection and conservation. Thus, the database makes available, for the public at large and the decision institutions, a list of the most vulnerable Romanian caves for their unique cave communities and species and a Red List of cave species, both in need of management measures.

### Additional information

**Figure 1.**
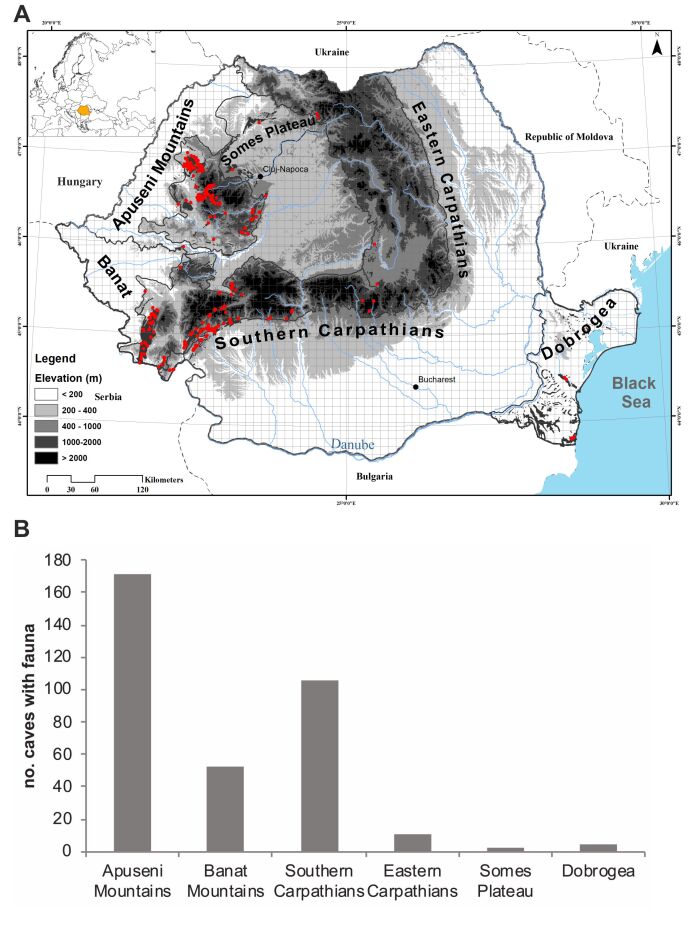
The caves (red dots) with fauna in the Romanian biospeleological regions (A) with the position of the country in Europe (upper left) and the number of caves with troglobionts/stygobionts in the different regions (B).

Romanian troglobionts and stygobionts are distributed mainly in caves of the Carpathian Mountains that can be separated into five main regions (Fig. 1); Apuseni Mountains (north-western Romania) and the Southern Carpathians, where most of the species were recorded, followed by Banat Mountains (south-western Romania), Eastern Carpathians and the Someș plateau (north of Apuseni Mountains). The formation of the Carpathians and paleogeographic evolution separated these regions after their colonisation by their cave fauna ancestors (Decu and Negrea 1969, Moldovan and Rajka 2007). Thus, each of these five main regions is populated mostly by taxa with a regional distribution. Dobrogea, the sixth Romanian biospeleological region, has three caves with strictly cave-adapted fauna, including Movile Cave that has more than 36 endemic cave species (Sarbu et al. 2019 and unpublished data).

**Figure 2.**
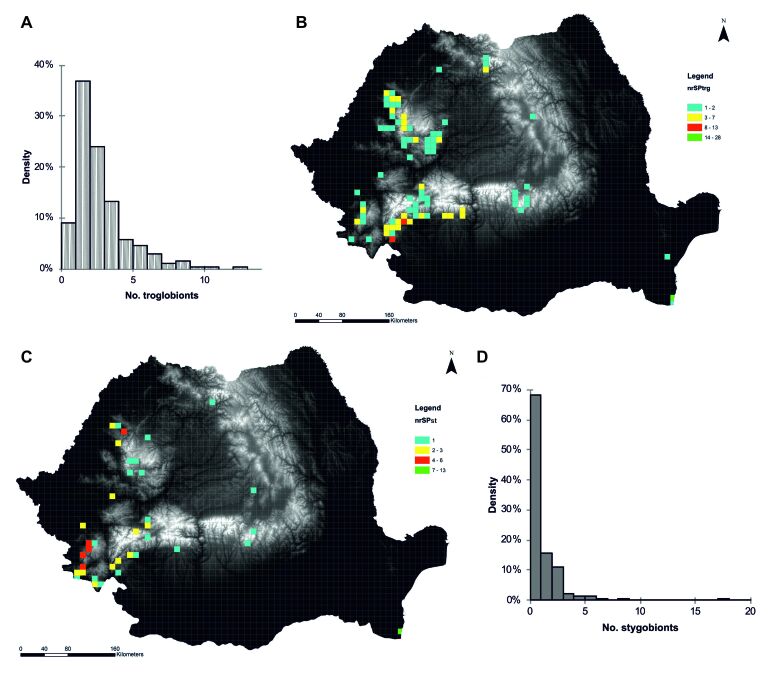
The frequency, distribution and abundance of troglobionts (nrSPtrg; A and B, respectively) and of stygobionts (nrSPst; D and C, respectively) in Romanian caves.

In our database, a total of 366 caves with troglobionts/stygobionts were registered (Fig. 2), but the number is higher because caves that belong to a larger underground system and caves which are located next to each other were registered as one entry. A total of 77 troglobionts and 96 stygobionts are in the database, not considering the undescribed species. About 40% of caves with fauna contain one troglobiont while about 70% of caves with fauna are populated by one stygobiont. For each cave, data on altitude (absolute & relative), temperature (degrees Celsius), precipitation (cumulative annual rainfall), vegetation cover, soil type, geology, protection status and threats were added. The environmental data are available upon request.

**Figure 3.**
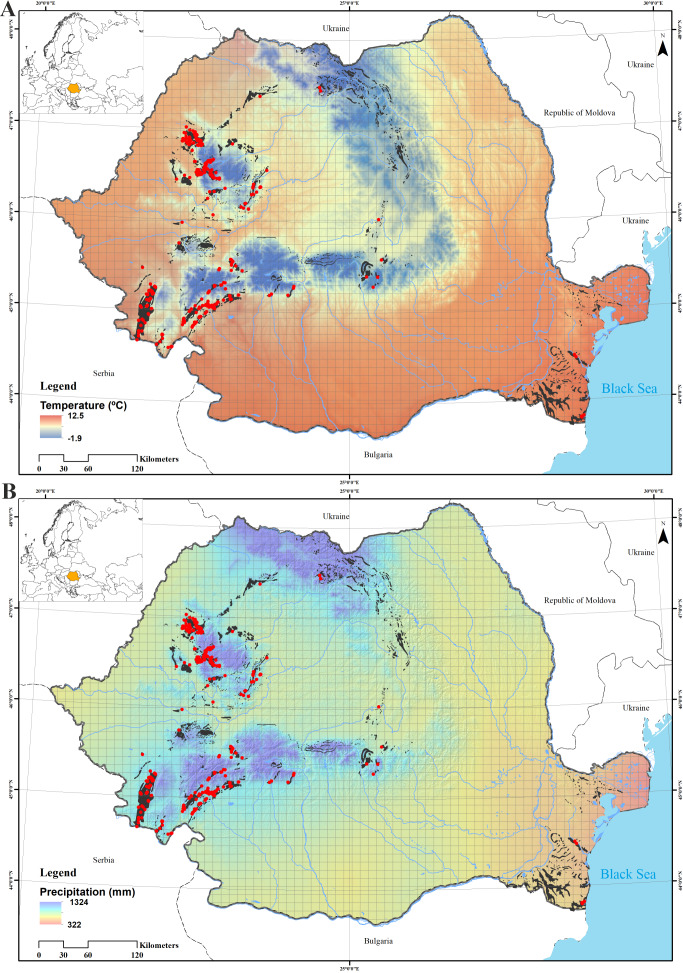
The annual mean temperature (A) and precipitation (B) in Romania, the red dots representing the caves with troglobionts/stygobionts. Climatic data from Fick and Hijmans (2017).

**Figure 4.**
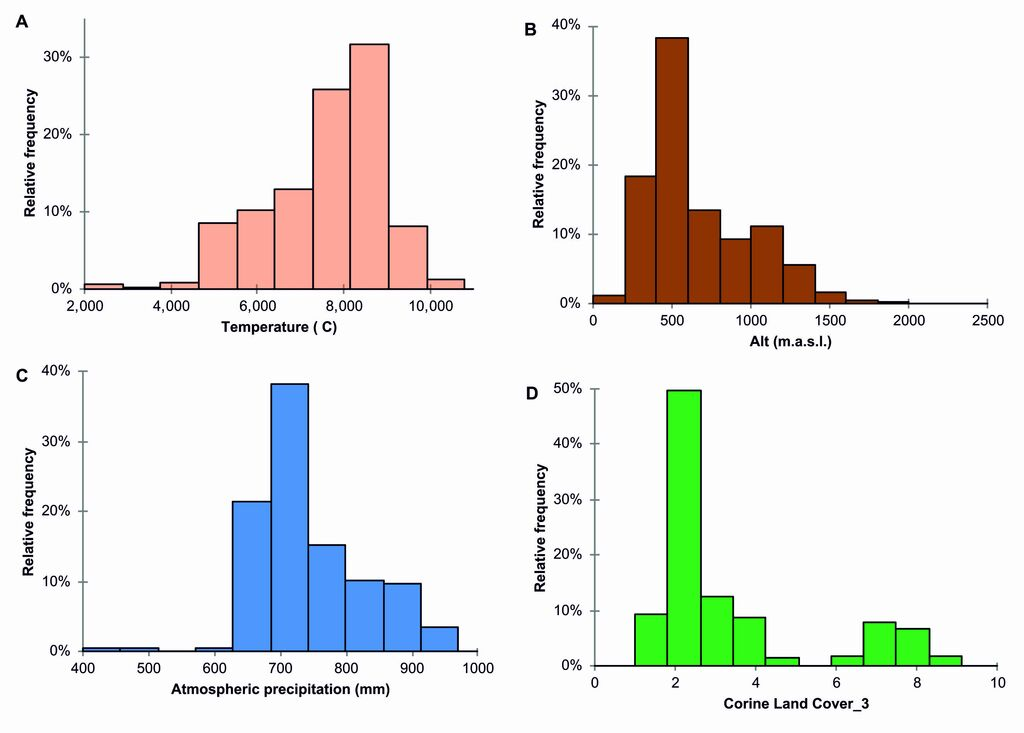
Figure 4. Relative frequency of troglobionts and stygobionts depending on the environmental characteristics of the surface. A. Temperature; B. Altitude; C. Precipitation; D. Vegetation: coniferous forest = 1, broad leaves forest = 2, mixed forest = 3, grassland = 4, woodland-shrubs = 5, bare rocks = 6, pastures = 7, agriculture land = 8, urban + mineral extraction = 9. Note that Movile Cave is not introduced in the analysis. Climatic data from Fick and Hijmans (2017), vegetation data from Academia RSR (1981)

**Figure 5.**
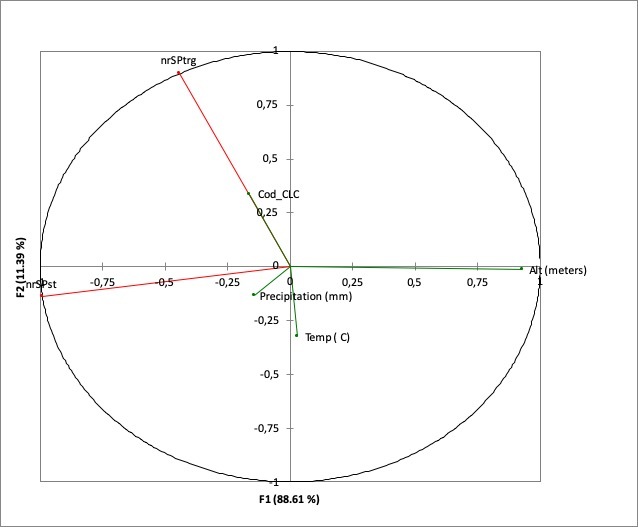
The Canonical Correlation Analysis on the relationship between environmental predictors (temperature, precipitation and altitude) and the number of troglobionts/stygobionts in Romanian caves shows no significant correlation. nSPtrg = number of troglobionts, nSPst = number of stygobionts.

According to statistical analysis, the richest caves are located at altitudes between 350 and 600 m a.s.l., where the mean annual temperature ranges between 7º and 9ºC (Fig. 3) and with a moderate amount of precipitation, 650-750 mm/year. Caves are particularly vulnerable and we assumed that their conservation depends on the integrity of surface ecosystems, translated into the integrity and quality of all its components - water entering underground, soil and vegetation cover. In our analyses, the most precise descriptor for caves with fauna was vegetation on the surface. Nearly 50% of the caves containing fauna are present in areas covered by broad-leaved forests (category 2 on D in Fig. 4). Vegetation on the surface was also a good predictor for the diversity of particular groundwater crustaceans groups (copepods and ostracods), especially in caves located at mid-elevation (Iepure et al. 2015). The soils, not represented in Fig. 4, were very diverse on the surface, with brown, rendzinic with limestone outcrops and the red with limestone outcrops as better represented above the caves with fauna. However, none of the considered environmental predictors correlated significantly with the presence of troglobionts/stygobionts (Fig. 5).

## Geographic coverage

### Description

Romania lies between latitudes 43º and 49º N and longitudes 20º and 30º E

## Taxonomic coverage

### Description

All invertebrates with strictly cave-adapted representatives in Romania (high-level classification from Zhang 2011). Undescribed species are not listed.

## Usage rights

### Use license

Creative Commons Public Domain Waiver (CC-Zero)

## Data resources

### Data package title

Biodiversity in Romanian caves with species and caves that are under threat or need special management measures.

### Resource link

Moldovan, Oana et al. (2020), Database of Romanian cave invertebrates, Dryad, Dataset, https://doi.org/10.5061/dryad.9ghx3fff9

### Number of data sets

4

### Data set 1.

#### Data set name

Troglobionts from Romanian caves

#### Number of columns

12

#### Description

Cave codes are from Goran (1982).

**Data set 1.** 

Column label	Column description
Genus	Genus name
Sub-genus	Sub-genus name
Species	Species name
Author, Year	Author and year of description
Family	Family name
Order	Order name
Class	Class name
Cave	Cave name
Code	Cave Code
Massif	Massif name
Mountain	Mountain name
Basin/Area	Basin or area name

### Data set 2.

#### Data set name

Stygobionts from Romanian caves

#### Number of columns

12

#### 

**Data set 2.** 

Column label	Column description
Genus	Genus name
Sub-genus	Sub-genus name
Species	Species name
Author, year	Author and year of description
Family	Family name
Order	Order name
Class	Class name
Cave	Cave name
Code	Cave code
Massif	Massif name
Mountain	Mountain name
Basin/Area	Basin or area name

### Data set 3.

#### Data set name

Red List of Romanian cave fauna

#### Number of columns

11

#### 

**Data set 3.** 

Column label	Column description
Genus/species	Genus and species
Author/year	Author and year of description
Family	Family name
Order	Order name
Class	Class name
Phylum	Phylum name
Kingdom	Kingdom name
IUCN Red List Category	IUCN Category
Geographic range	Geographic distribution
Threats	Description of threats
Conservation action	Conservation measures

### Data set 4.

#### Data set name

Hotspot and coldspot caves in Romania

#### Number of columns

5

#### Description

Classes of cave protection in Romania and their main characteristics. A = caves of exceptional value, which by their scientific or unique resources, are representative for national and international heritage; B = caves of national importance, distinguished by size, scarcity of resources and touristic potential; C = caves of local importance, protected for their geological, landscaping, hydrological, historical, biodiversity significance, touristic potential or their dimensions; D = small or medium caves without special value, but important for the regional geology, biodiversity and evolution that must be preserved and protected from pollution or destruction (Moldovan 2019).

**Data set 4.** 

Column label	Column description
Cave	Cave name
No. species/no. endemics	Number of species and number of endemic species for this cave
Protection status	Protection status according to Romanian legislation
Threats	Description of threats
Massif	Massif name

## Additional information

The high-impact paper on hotspots published by Myers (1988) opened a new era in nature conservation. While it is impossible to conserve all biodiversity on Earth - or in caves - the idea was to focus the conservation efforts on selected biodiversity hotspots. However, the optimal conservation network should include the so-called coldspots represented by areas with rare species (Kareiva and Mareiva 2003), as is the case for most of the situations in caves. The loss of biodiversity in terrestrial and aquatic ecosystems, where most species are endemic and rare, can irreversibly disrupt the integrity of the ecosystems and, consequently, the possibility to preserve functions and evolutionary processes (Bøhn and Amundsen 2004). This idea was sustained for deep marine environments by Price (2002) and is the perfect description of the cave ecosystems, with caves defined as hotspots or coldspots or even a mixture of hotspots and coldspots (https://airtable.com/shr9YPMh9gybZG7fu).

Currently, there is no conservation status for the subterranean invertebrate species in Romania, since none of the species is included in national lists of protected species or in the current IUCN list. With the obtained information, we propose a list of coldspot-caves that host rare species, endemic species for single caves and highly biodiverse in troglobionts/stygobionts and a Red List of Romanian strictly-adapted cave species Most of the caves in this List are included in class A of protection (44%) and 14% in A/B or B/A (Moldovan 2019), the highest levels of protection for Romanian caves. Such classes of protection for Romanian caves are limiting the use of the caves for human and economic activities. However, in a large number of caves, we identified four main activities that threaten their fauna: intensive organised speleological tourism (22%), pollution (5%) and water extraction (two cases). These were identified by taking into account reports from cavers while also noticing the total lack of fauna in known spots along caves during repeated visits over several years. All identified caves should be considered as good candidates for protection and in need of an integrated and updated monitoring programme (for air/water quality and assessment of troglobionts/stygobionts communities) where touristic or other activities should be limited. In several caves, additional protection of the reception hydrographic basin and moderate land use, with no forest cuttings or other human activities at the surface with an impact on the subterranean environment, should be applied. For the tentative Red List of troglobionts and stygobionts, we selected only the species whose distribution is restricted to one cave and one-few spots inside the cave, with known small populations and which are vulnerable to existing threats.

Most of the gaps in the database are linked to both uneven sampling efforts, with extensive sampling near the biospeleological Romanian centres of Cluj and București and uneven sampling of some of the groups, such as Coleoptera. Errors are also due to the lack of recent assessment of the status of most of the caves with fauna, i.e. their physical and climatic integrity, invertebrates presence or population sizes. Nevertheless, this database can and should be the starting point for any decision linked to the use of cave habitats for touristic or other purposes.
